# The first decade of the Danish Palliative Care Database: improvements and ongoing challenges in the quality and use of specialised palliative care

**DOI:** 10.2340/1651-226X.2024.28515

**Published:** 2024-05-02

**Authors:** Mathilde Adsersen, Maiken Bang Hansen, Mette Asbjoern Neergaard, Per Sjøgren, Mai-Britt Guldin, Mogens Groenvold

**Affiliations:** a Palliative Care Research Unit, Department of Geriatrics and Palliative Medicine, Bispebjerg and Frederiksberg Hospital, University of Copenhagen, Copenhagen, Denmark; b Palliative Care Unit, Department of Oncology, Aarhus University Hospital, Aarhus, Denmark; c Department of Clinical Medicine, Aarhus University, Aarhus, Denmark; d Section of Palliative Medicine, Department of Oncology, Rigshospitalet, Copenhagen University Hospital, Copenhagen, Denmark; e Research Unit for General Practice, Aarhus and Institute for Public Health, Aarhus University, Aarhus, Denmark; f Department of Public Health, University of Copenhagen, Copenhagen, Denmark

**Keywords:** Palliative care team, hospice care, database, quality indicator, cancer, non-cancer

## Abstract

**Background:**

Danish Palliative Care Database comprises five quality indicators: (1) Contact with specialised palliative care (SPC) among referred patients, (2) Waiting time of less than 10 days, (3) Proportion of patients who died from (A) cancer or (B) non-cancer diseases, and had contact with SPC, (4) Proportion of patients completing the patient-reported outcome measure at baseline (EORTC QLQ-C15-PAL), and (5) Proportion of patients discussed at a multidisciplinary conference.

**Purpose:**

To investigate changes in the quality indicators from 2010 until 2020 in cancer and non-cancer patients.

**Patients/material:**

Patients aged 18+ years who died from 2010 until 2020.

**Method:**

Register-based study with the Danish Palliative Care Database as the main data source. Indicator changes were reported as percentage fulfilment.

**Results:**

From 2010 until 2020, the proportion of patients with non-cancer diseases in SPC increased slightly (2.5–7.2%). In 2019, fulfilment of the five indicators for cancer and non-cancer were: (1) 81% vs. 73%; (2) 73% vs. 68%; (3A) 50%; (3B) 2%; (4) 73% vs. 66%; (5) 73% vs. 65%. Whereas all other indicators improved, the proportion of patients waiting less than 10 days from referral to contact decreased. Differences between type of unit were found, mainly lower for hospice.

**Interpretation:**

Most patients in SPC had cancer. All indicators except waiting time improved during the 10-year period. The establishment of the Danish Palliative Care Database may have contributed to the positive development; however, SPC in Denmark needs to be improved, especially regarding a reduction in waiting time and enhanced contact for non-cancer patients.

## Introduction

Pressure on the health care system and limited resources make it important to ensure the best possible admittance to and quality of important health care services, including palliative care. Internationally, increased focus is being placed on data about the development and the quality of palliative care. National palliative care registers (or databases) have been established in several countries including Denmark (Danish Palliative Care Database) [1], Sweden (The Swedish Register of Palliative Care) [2], Germany (Hospice and Palliative Care Register) [3], the United States (National Palliative Care Registry, recently (2020) united with other organisations called Palliative Care Quality Collaborative) [4], Australia (Palliative Outcomes Collaboration) [5], and more recently in the United Kingdom (Palliative Care Outcomes Registry) [6]. The target populations, variables, and quality indicators vary between countries.

In Denmark, the first specialised palliative care (SPC) units (i.e., hospital-based palliative care teams/units and hospice) were established in the 1990s [7, 8], and in the following 20 years little was known about the quality of SPC due to a lack of systematic sampling of national data. However, a founding board of a national clinical quality database, the Danish Palliative Care Database, was established in 2007, and in 2010 registration of patients in the database began. Since then, national data about all patients referred to and/or admitted to SPC have been collected at the end of each SPC trajectory, allowing for annual reporting of five quality indicators (Table 1) at a national as well as at a local unit level [9].

**Table 1:** The definitions and standards of the five indicators.

Indicators	Definition of the indicators (for patients aged 18+ years	Standard
Indicator 1 – Proportion of eligible patients referred to specialised palliative care who had contact	$\frac{Number\;of\;patients\;who\;had\;contact\;with\;specialised\;pallaitive\;care}{\begin{array}{l} Number\;of\;referred\;patients\;who\;fullfilled\;the\;referral\;criteria*\;for\;specialised\;palliative\;care,\;were\\ suitable\;for\;treatment**,\;did\;not\;regret\;being\;in\;contact\;and\;were\;not\;in\;contact\;with\;another\;specialised\;pallaitive\;care\;unit \end{array}}*100$	80%
Indicator 2 – Proportion of patients with contact to specialised palliative care waiting a maximum of 10 days from referral to contact	$\frac{Number\;of\;patients\;who\;had\;contact\;with\;specialised\;palliative\;care\;within\;10\;days\;from\;referral}{Number\;of\;patients\;who\;had\;contact}*100$	90%
Indicator 3A – Proportion of patients with cancer who had contact with specialised palliative care	$\frac{Number\;of\;patients\;with\;cancer\;who\;had\;contact\;with\;specialised\;palliative\;care}{Number\;of\;deaths\;from\;cancer}*100$	35%
Indicator 3B – Proportion of patients with non-cancer diseases who had contact with specialised palliative care	$\frac{Number\;of\;patients\;with\;diseases\;other\;than\;cancer\;who\;had\;contact\;with\;specialised\;palliative\;care}{Number\;of\;deaths\;from\;other\;diseases\;than\;cancer}*100$	#
Indicator 4 – Proportion of patients who had contact and completed patient-reported outcome measures (EORTC QLQ-C15-PAL)	$\frac{Number\;of\;patients\;who\;completed\;the\;EORTC\;questionnaire\;at\;contact\;\left(i.e.,\;3\;days\;before\;or\;on\;the\;day\;of\;the\;first\;contact\right)}{Number\;of\;patients\;who\;had\;contact}*100$	50%
Indicator 5 – Proportion of patients with contact to specialise palliative care who were discussed at a multidisciplinary conference	$\frac{Number\;of\;patients\;discussed\;at\;a\;multidisciplinary\;conference\;with\;staff\;from\;at\;least\;four\;different\;disciplines}{Number\;of\;patients\;who\;had\;contact}*100$	80%

*Fulfilment of the referral criteria was based on a palliative care specialist’s evaluation. From August 2017, national referral criteria were developed and are as follows: Patients with a life-threating disease and complex palliative symptoms or problems.

**Assessment of the clinician in the specialised palliative care unit who received the referral of the patient

# No standard is defined for indicator 3B..

The aims of this study were to investigate changes in the quality indicators of the Danish Palliative Care Database of SPC in Denmark during the first 10 years from 2010 until 2020. Further, to compare trends of cancer and non-cancer patients in relation to hospital-based palliative care teams/units and hospices.

## Methods and material

### Setting

In Denmark (5.8 million inhabitants), SPC is delivered by hospital-based institutions (palliative care teams/units) (2019, *n* = 25) and hospices located outside the hospital as independent units financed by the regions (2019, *n* = 19) [9]. Most patients treated by palliative care teams/units are outpatients, whereas hospices mainly treat inpatients. Physicians in both primary and secondary health care sectors can refer to SPC, which is free of charge because of a tax-financed health care sector.

### Danish Palliative Care Database

The purpose of the Danish Palliative Care Database is: ‘to monitor, evaluate, and improve the clinical quality of specialised palliative care (SPC) (i.e., the activity of hospital-based palliative care teams/departments and hospices) in Denmark’ [1]. Since 1 January 2010, it has been mandatory for all SPC units in Denmark to report demographic, social, and clinical data about all patients referred to SPC to the Danish Palliative Care Database [1]. The Danish Palliative Care Database covers SPC only. The patient completeness, determined for each SPC service by cross-checking reported patients against the National Patient Register [10], has been high, increasing from 95.7% in 2010 to 100.0% from 2012 until 2020 [9].

The five quality indicators of the Danish Palliative Care Database measure: (1) Contact with SPC among referred patients, (2) Waiting time (proportion of patients waiting less than 10 days), (3) Proportion of patients who died from (A) cancer or (B) non-cancer diseases, (4) Proportion of patients completing the patient-reported outcome measure at baseline (EORTC QLQ-C15-PAL), and (5) Proportion of patients discussed at a multidisciplinary conference with at least four types of health care professionals present. The definitions of the five quality indicators of the Danish Palliative Care Database are described in Table 1. For each indicator, except indicator 3B, a standard (goal) was developed, based on the literature, assessment, and consensus in cooperation with the Danish Palliative Care Database board, clinicians from the SPC units, and experts in palliative medicine [1, 11].

### Data sources

For indicators 1, 2, 4, and 5, the Danish Palliative Care Database was the only data source and contributed with data about the date of referral; whether the referral criteria were fulfilled; contact with SPC including date of first contact, type of unit, diagnosis; completion of the EORTC QLQ-C15-PAL questionnaire [12], and whether discussed at multidisciplinary conferences.

The data sources for indicators 3A and 3B also included the Nordic Cancer Registries [13] and the Register of Causes of Death with data about cause of death [14].

### Study population

The study population included all patients aged 18+ years who were referred to SPC from 2010 and who died from 1 January 2010 through 31 December 2019. The indicators for 2010 were lower than the following years as data for patients who died in 2010 but were referred to SPC before 2010 were not included in the database. Indicators 2, 4, and 5 contain patients who were admitted to SPC. For indicators 3A and 3B, the patients were divided into patients who died from cancer (Nordic Cancer Registries) and patients who died from diseases other than cancer (Register of Causes of Death), respectively (Table 1).

### Statistics

The change in the five quality indicators in Denmark from 2010 until 2020 was investigated for patients with cancer and non-cancer. For indicators 1, 2, 4, and 5, the development was further investigated for contact with hospital-based palliative care teams and hospices. The definitions of indicators 3A and 3B (see Table 1) include SPC overall, and therefore, a division into type of institution was not relevant. The results of the five quality indicators were reported as percentage fulfilment in graphs along with the standard for each indicator.

## Results

From 2010 until 2020, 145,006 referrals of patients who were deceased by the end of the decade were registered in the Danish Palliative Care Database. Of the 145,006 referrals, 81% were deemed eligible for contact with SPC by SPC unit, and 94,289 (65%) of the 145,006 referred patients had contact. Gender distribution was equal, and the mean age was 70.3 years (range: 18–109 years) (data not shown).

**Figure 1:**
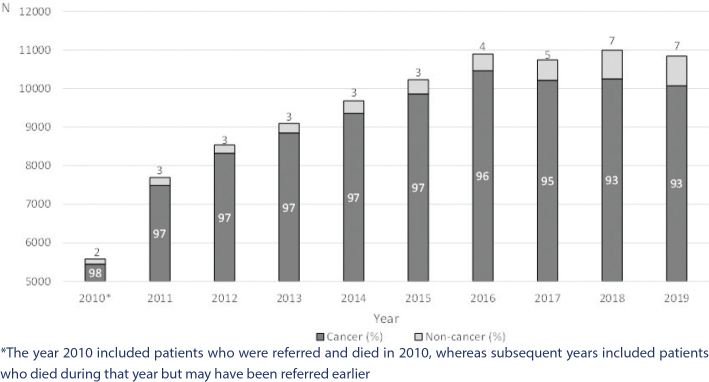
Number of patients who had contact with specialised palliative care from 2010 to 2020 and annual number of cancer and non-cancer patients according to the Danish Palliative Care Database.

A low number of patients was seen in 2010 because this year included patients who were referred and died in 2010, whereas subsequent years included those who died during that year but may have been referred earlier. The proportion of non-cancer patients who had contact was 4% across the entire period (2010 until 2020) and increased from 2 to 7% (Figure 1).

### Indicator 1

**Figure 2:**
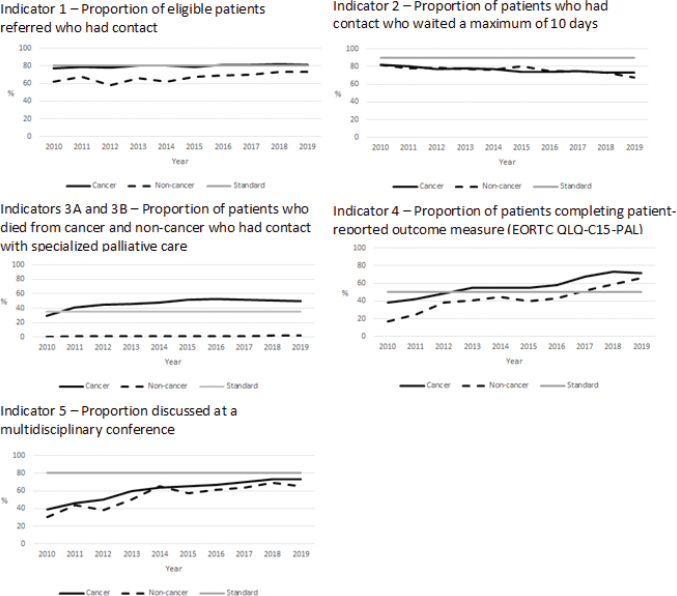
Indicators 1–5 for cancer and non-cancer patients from 2010 until 2020. The year 2010 included patients who were referred and died in 2010, whereas subsequent years included patients who died during the year but may have been referred earlier.

**Figure 3:**
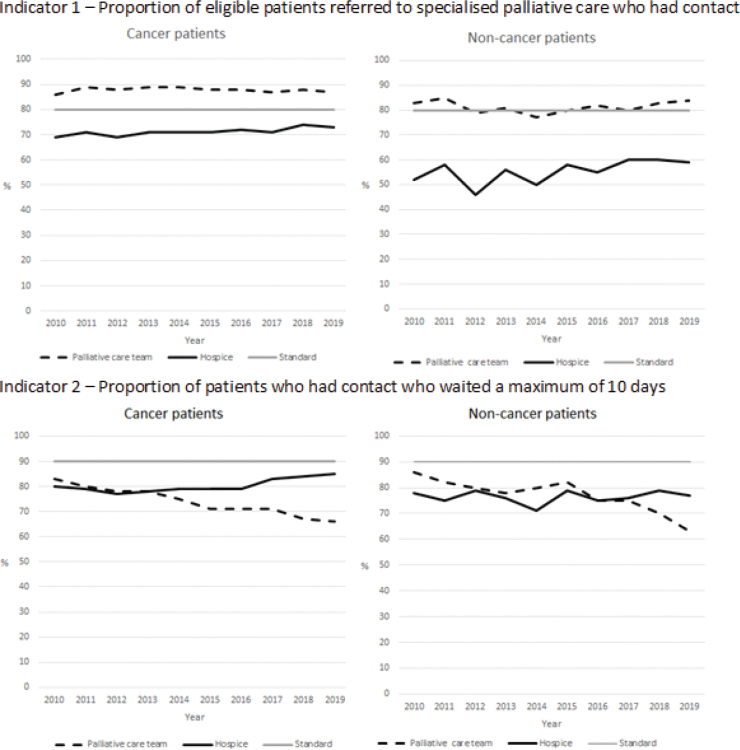
Indicators 1 and 2 for cancer and non-cancer patients in relation to palliative care team and hospice from 2010 until 2020. The year 2010 included patients who were referred and died in 2010, whereas subsequent years included patients who died during that year but may have been referred earlier.

**Cancer:** In 2010, 77% of the eligible patients with cancer referred to SPC had contact with specialised palliative . The proportion increased slightly to 80% in 2013, and was stable around that level until 2019 (81%) (Figure 2). Across the decade, a higher proportion of referred patients had contact with hospital-based palliative care teams than to hospices (2019: 87 vs. 73%) (Figure 3).

**Non-cancer:** The proportion of patients who had contact with SPC was lower for non-cancer patients, as 62% of the referred patients had contact with SPC in 2010 and 73% had contact in 2019, the increase taking place from 2015 (Figure 2). The proportion who had contact with SPC was higher for those referred to palliative care teams than for those referred to hospices (2019: 84 vs. 59%) (Figure 3).

The standard for indicator 1 (80%) was fulfilled for patients with cancer and non-cancer for palliative care teams but not for hospices (Figure 3).

### Indicator 2

**Cancer:** The proportion of patients waiting a maximum of 10 days decreased from 2010 until 2020 for the palliative care teams, but increased for hospices, especially from 2016 onwards (Figure 3). Since 2014, the proportion waiting a maximum of 10 days was higher for hospices than for palliative care teams (in 2019: 85% vs. 66%) (Figure 3).

**Non-cancer:** A decrease from 82 to 68% in the proportion of patients waiting a maximum of 10 days from 2010 until 2020 was found for non-cancer patients referred to palliative care teams (Figure 3); however, for hospices it varied between 71 and 79%. In 2019, the figures for hospices and palliative care teams were 77 and 63%, respectively (Figure 3).

The standard (90%) was never fulfilled overall or in the sub-groups.

### Indicator 3

**Cancer:** The proportion of patients who died from cancer and had contact with SPC increased until 2016 (29–53%; the 29% in 2010 may be explained by the restrictions in the first year of the database) and then decreased by 1% per year to 50% in 2019 (Figure 2). The standard for indicator 3 (35%) has been fulfilled since 2011 (Figure 2).

**Non-cancer:** The proportion of patients dying from non-cancer diseases who had contact with SPC increased slightly from 0% in 2010 to 1% in 2011, and to 2% in 2018 and 2019 (Figure 2). A standard was not developed for non-cancer patients.

### Indicator 4

**Figure 4:**
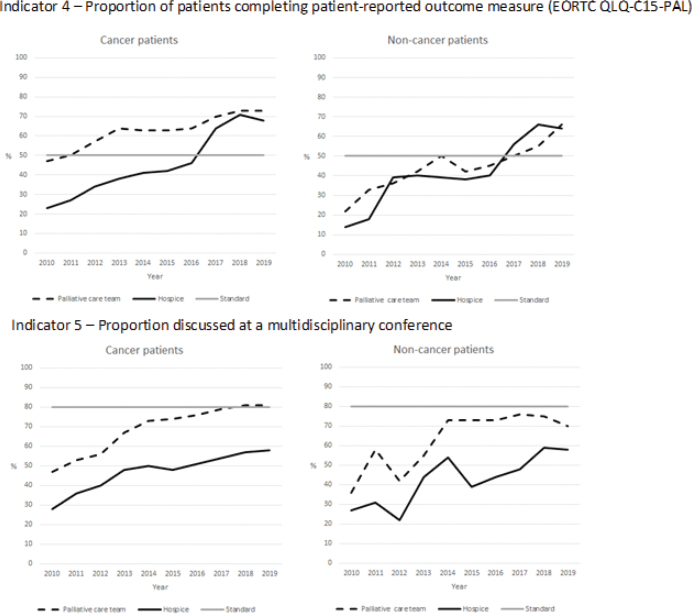
Indicators 4 and 5 for cancer and non-cancer patients in relation to palliative care team and hospice from 2010 to 2020. The year 2010 included patients who were referred and died in 2010, whereas subsequent years included patients who died during that year but may have been referred earlier.

**Cancer:** In 2010, 38% of the patients who had contact with SPC completed the quality-of-life questionnaire, EORTC QLQ-C15-PAL; however, this increased to the highest level in 2018/19 (73%) (Figure 2). The increase was seen for both types of units, and in 2019 the proportion completing the EORTC QLQ-C15 questionnaire was 73% for palliative care teams and 68% for hospices (Figure 4).

**Non-cancer:** An increase in the proportion completing the EORTC QLQ-C15-PAL was also found for non-cancer patients, rising from 17% in 2010 to 66% in 2019 (Figure 2). In 2019, similar proportions completing the EORTC QLQ-C15-PAL were found for palliative care teams and hospices (Figure 4).

From 2017, the indicator values for all subgroups were above the standard at 50%.

### Indicator 5

**Cancer:** The proportion of patients with cancer discussed at a multidisciplinary conference within 4 weeks after contact increased from 39 to 73% from 2010 until 2020 (Figure 2). A higher proportion of cancer patients in palliative care teams than in hospices was discussed at a multidisciplinary conference during the observation period (in 2019: 81% vs. 58%) (Figure 4).

**Non-cancer:** A similar pattern was seen for non-cancer patients, rising from 30% in 2010 to 65% in 2019; however, the proportion varied and was highest in 2018 (69%) (Figure 2). Similar to cancer patients, a higher proportion of non-cancer patients in palliative care teams than in hospices was discussed at a multidisciplinary conference during the observation (in 2019: 70% vs. 58%) (Figure 4).

In 2019 all subgroups, except cancer patients in palliative care teams (81%), were below the standard at 80%.

## Discussion

Data from the 10-year period show that the proportion of non-cancer patients who had contact with SPC remained low, but increased slightly during the period. For cancer and non-cancer patients, all indicators improved during the 10-year period, except indicator 2 (waiting time). All indicators were lower for non-cancer patients than for cancer patients, and marked differences between type of unit (mainly lower for hospices than for palliative care teams) were found in relation to several of the indicators.

The results for indicators 1–3 (contact among referred, waiting time and contact for patients who died from cancer or non-cancer diseases) indicate that the capacity of SPC increased from 2010 to 2015 in Denmark, and then stagnated [15]. In Denmark, the capacity for SPC is only around half the capacity of international recommendations and standards [15–17]. For cancer patients, the contact (indicator 3A) increased from 2010 until 2015, alongside the expansion of the capacity [15]; however, from 2016 a slight decrease in contact was observed in the data from the Danish Palliative Care Database, whereas a slight increase was observed for non-cancer patients from 2018. In December 2017, the Danish Health Authority published new recommendations for palliative care in Denmark [18], with an increased focus on patients with diseases other than cancer, stating that the needs of patients with diseases other than cancer seem to be of the same magnitude as patients with cancer [19]. These new recommendations for palliative care can be a part of the explanation for the (slight) increase in contact with SPC for non-cancer patients to 2% in 2018. However, the recommendations were not followed by economic funding, and therefore, SPC may not have been developed structurally or clinically to ensure SPC to patients other than those with cancer. The decrease in contact for patients with cancer in the same period (2017–18) may reflect that the capacity remained stable in spite of a rapidly expanding patient population for SPC including cancer and non-cancer patients [18]. Other marked health care reforms or changes in practice have not taken place in the Danish health care system during the study period.

The lower proportion of referred eligible patients who had contact with hospices than with palliative care teams could be explained by the organisation, as it may be more feasible for a hospital-based, outgoing palliative care team to include one more patient than for a hospice where an available bed is needed. The lower contact of non-cancer patients with hospices may reflect a perceived lack of knowledge to take care of non-cancer patients or a tradition, where some hospices have only accepted patients with cancer (it was one of the referral criteria at some SPC units until national criteria were introduced at the end of 2017).

The increase in waiting time for palliative care (indicator 2) is problematic for patients with severe, unrelieved symptoms or a short remaining lifetime. Even though the capacity has increased during the 10-year period, we can conclude that the increase was not sufficient to meet the increasing demand from cancer patients and non-cancer patients and resulted in longer waiting times.

Palliative care is often separated into SPC and general palliative care [18, 20]. However, these two sectors are closely connected [21]. The low capacity for SPC in Denmark means that the capacity for general palliative care needs to be increased to meet the palliative care needs of the population. Thus, at a national level the focus in Denmark is to increase the capacity for and quality of general palliative care [22]. As an example of this, the national cancer quality organisation ‘Danish Multidisciplinary Cancer Groups’ established the ‘Committee for Cross-Disciplinary Palliative Care Collaboration’ in 2019 in order to improve palliative care in oncology via quality indicators and clinical guidelines for palliative care needs assessment and palliative care [23]. However, there is still no national measurement of the amount, distribution, or quality of general palliative care [24]. It is therefore not possible to investigate the development of general palliative care in relation to the stagnant development of SPC. Another example is the lack of palliative care as a medical speciality in Denmark.

The increase in the proportion completing the questionnaire, EORTC QLQ-C15-PAL, at admission (indicator 4) and the proportion being discussed at multidisciplinary conferences (indicator 5) could be explained by the endeavours of the Danish Palliative Care Database. The annual reports from the Danish Palliative Care Database give the results of the indicators by unit. These results are given wide attention and are often accompanied by local and regional audits that may have increased the focus on these opportunities for improvement. Besides the annual reports, each unit can follow the development of the indicators by way of monthly digital data which can identify problems at an early stage.

The marked increase in the proportion who completed the quality of life questionnaire, EORTC QLQ-C15-PAL (indicator 4) from 2016 until 2019 could also be explained by a large national improvement project in SPC which was carried out from February 2017 to July 2019 [25]. The improvement project aimed to promote implementation of clinical guidelines, and for symptoms above certain thresholds in the EORTC QLQ-C15-PAL, the clinicians had to report whether care according to guidelines was delivered [25, 26]. This improvement project likely increased the focus nationally on completing the EORTC QLQ-C15-PAL.

The difference in the indicators between the two types of specialist palliative care (palliative care teams and hospices) may reflect their patient populations. The lives of patients with contact to hospice are shorter than patients in contact with a palliative care team, median 20 days vs. median 66 days [9]. This may explain the lower proportion discussed at multidisciplinary conferences and the number of patients with cancer who completed the EORTC QLQ-C15-PAL. Another explanation could be a difference in the organisation of the work in the different types of units and/or limited access to psychologists, physiotherapists, and/or social workers which would decrease thereby the possibility to have frequent multidisciplinary conferences, for example, more than once per week.

Because of the low capacity for SPC in Denmark and longer waiting times, it is necessary to ensure that the patients in contact with SPC are those who need it the most. An important tool in that process will be implementation of the nationally developed patient-reported outcome questionnaire for identification of palliative needs among patients with advanced disease [27], and further, to ensure data availability regardless of health care sector. Moreover, we need to ensure that health care professionals in SPC play a key role in increasing the quality of general palliative care, for example, through the palliative care education of other health care professionals.

### Strengths and limitations

The main strength of this study is that it is based on an almost complete national sample of data from palliative care teams and hospices, which minimises the risk of selection bias. These data have made it possible to monitor the national development over 10 years, which can be difficult to obtain in palliative care. It is likely that the referred non-cancer patients are not representative for all non-cancer patients with a life-threatening disease, but they are representative for those who are referred to SPC. In this study, it has been possible to investigate the five quality indicators of palliative care in the Danish Palliative Care Database; however, not all relevant aspects of palliative care are included in these indicators. It is a limitation of the study that it was not possible to investigate over time whether there has been a development in the number and type of SPC contacts (e.g., inpatient care) per patient, as each unit only enters data about the first contact. Another limitation is that data for the indicators for the year 2010 are limited to patients who were referred and died in 2010. The number of patients included in the indicators for 2010 will therefore be lower than the real number of patients, as patients referred to SPC in 2009 and died in 2010 will not be included in the Danish Palliative Care Database. Data registration in the Danish Palliative Care Database began in 2010 and was not mandatory before 2010.

### Generalisability

The results obtained with use of data from the first decade of the Danish Palliative Care Database show the possibilities to measure and follow data on patients in SPC. In other countries, national databases on palliative care may also be an important and relevant tool to monitor the development in palliative care and thereby improve it.

## Conclusion

In Denmark, patients who are in contact with SPC are mainly those suffering from a cancer disease, and the proportion with non-cancer diseases has only increased slightly. All quality indicators in the Danish Palliative Care Database have improved during the period from 2010 until 2020, except waiting time. Pronounced differences in relation to diagnosis (with lower quality for non-cancer patients) and type of unit were found. We estimate that the establishment of the Danish Palliative Care Database has contributed to a positive development; however, SPC needs to be further developed in Denmark especially with regard to contact and waiting time, particularly for non-cancer patients. Currently, the board of the Danish Palliative Care Database is developing new quality indicators on symptom relief using symptomatology measurement (EORTC QLQ-C15-PAL) which will allow the database to extend future monitoring.

## Authorship

MA and MBH were the major contributors in analysing data. MA and MGr were the major contributors in writing the manuscript. MAN, PE, and MGu were part of the data collection and/or contributed with clinical comments to the manuscript, especially in relation to the Introduction and Discussion sections. All authors have given their approval of the final version of the manuscript.

## Data Availability

Data are available at Danish Palliative Care Database, Regionernes Kliniske Kvalitetsprogram (RKKP, www.rkkp.dk ). Restrictions apply to the availability of these data.
